# Dynamic crosstalk between HSCs and liver microenvironment: multicellular interactions in the regulation of liver fibrosis

**DOI:** 10.3389/fcell.2025.1635763

**Published:** 2025-07-21

**Authors:** Luping Wang, Yi Huang, Jingrong Chen, Jialu Gao, Sihan Chen, Mingqi Zhao, Jiguo Lin, Shunqing Zhou, Yannan Shen, Yunyun Cheng

**Affiliations:** ^1^NHC Key Laboratory of Radiobiology, College of Public Health, Jilin University, Changchun, China; ^2^Department of Obstetrics and Gynecology, The Second Hospital of Jilin University, Changchun, Jilin, China

**Keywords:** liver fibrosis, hepatic stellate cells, cell communications, hepatic immune cells, intercellular signal transduction

## Abstract

Liver fibrosis is induced by persistent stimulation of various factors, resulting from complex multicellular interactions and multifactorial networks. Without intervention, it can progress to cirrhosis and even liver cancer. Current understanding suggests that liver fibrosis is reversible, making it crucial to explore effective therapeutic strategies for its alleviation. Although the activation and proliferation of hepatic stellate cells (HSCs) play a pivotal role in liver fibrosis, the importance of hepatocytes, cholangiocytes, liver sinusoidal endothelial cells (LSECs) and immune cells cannot be ignored, the interactions of these cells with HSCs are worth discussing. Therefore, based on the diversity of cell composition in the liver organ, this review summarizes the impact of the parenchymal and nonparenchymal hepatic cells on liver fibrosis, including hepatocytes, cholangiocytes, hepatic macrophages, T cells, NK cells, B cells and LSECs, as well as the fibroblast subpopulations. And further discussed the interactions of these cells with HSCs and illustrated intercellular signal transduction among these cells in contributing to liver fibrosis. Clarifying the roles and interactions of various cells in the development of liver fibrosis will be helpful to explore effective strategies for the treatment of liver fibrosis.

## 1 Introduction

Liver fibrosis is induced by the chronic and persistent stimulation of various factors such as liver injury, resulting in an imbalance between damage and repair. The activation of hepatic stellate cells (HSCs) is considered to be one of the key events, which in turn contributes to the accumulation of extracellular matrix (ECM). Liver fibrosis does not occur as a result of the independent action of individual cells, but as a complex multicellular network. Although the activation and proliferation of HSCs play a pivotal role in liver fibrosis, the importance of hepatocytes, cholangiocytes, liver sinusoidal endothelial cells (LSECs) and immune cells cannot be ignored.

It has been shown in the early state of liver injury, the damaged or dead hepatic parenchymal cells (hepatocytes) are able to release nucleotides, reactive oxygen species (ROS), Hh ligands, and damage-associated molecular modules (DAMPs), which interact with HSCs in the interstitium (Tsuchida and Friedman, 2017). In addition to HSCs, all nonparenchymal cells in the liver also play a role in the occurrence of hepatic fibrosis mediated by HSCs through their secretory substances. Of which, cholangiocyte are involved in biliary fibrosis, which is related to hepatic fibrosis. Various factors like infection, cholestasis and ischemia can stimulate cholangiocyte activation (O'Hara et al., 2017), leading to ductular reaction (DR) and interact with hepatic fibrosis. Macrophages are considered to be central regulators among the immune cells associated with liver fibrosis. Macrophages can regulate the homeostasis of hepatic fibrosis and even cause regression of hepatic fibrosis by secreting matrix-degrading enzymes such as matrix metalloproteinases (MMP) (Tacke and Zimmermann, 2014). Macrophages have a heterogeneous phenotype because they owns highly plasticity in their response to local environmental stimuli. The liver is the site of accumulation of many innate lymphocyte populations (ILTCs), including natural killer cells (NK), CD56 (+) T cells, natural killer T cells (NKT), γδ T cells, and mucosa-associated invariant T cells (MAIT), the latter three of which belong to unconventional T cell subsets. The heterogeneity of T cells and the wide variety of effectors which include various ligands and cytokines, play an important role in the development of liver fibrosis, and they are involved in a close and complex crosstalk with HSCs activation. B cells are a key component of the adaptive immune system, providing specific and long-lasting protection against a large number of potential pathogens through the production of highly diverse and specific antibodies (Visentini et al., 2022), while they can also progressively exacerbate the disease through the secretion of pro-inflammatory factors, antibodies, and the recruitment of other cells. In the human liver, B cells comprise only 8% of the intrahepatic lymphocyte population (Patel et al., 2021). Although there are only a small number of B cells, they also are important in the development of liver diseases. In injured liver, the differentiation of LSECs into capillarization initiates and the inhibition of HSCs activation is reduced to a lesser extent. Depending on the injury, LSECs are able to promote either hepatocyte regeneration or liver fibrosis (Xie et al., 2012).

**FIGURE 1:**
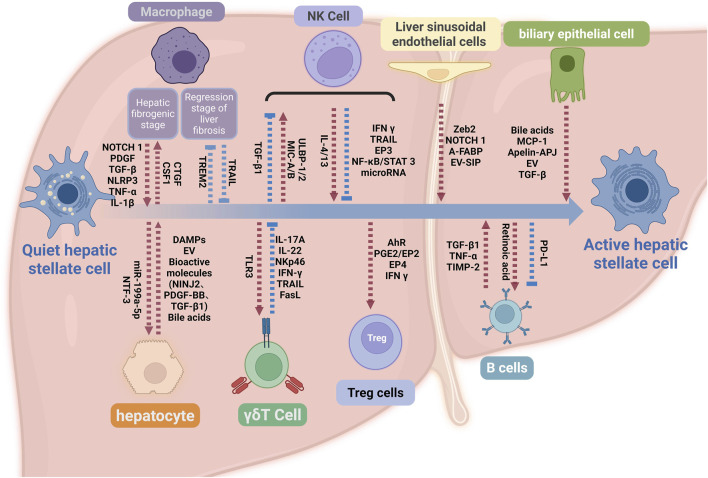
Overview of the interactions of various hepatic cells in the development of liver fibrosis.

The occurrence of liver fibrosis is a complex process involving multiple cells. Considering the diversity of cell composition in the liver organ, clarifying the roles and interactions of various cells in the development of liver fibrosis is helpful to explore effective strategies for the treatment of liver fibrosis (Figure 1).

## 2 Interaction of HSCs with hepatocytes

### 2.1 Hepatocytes

**FIGURE 2:**
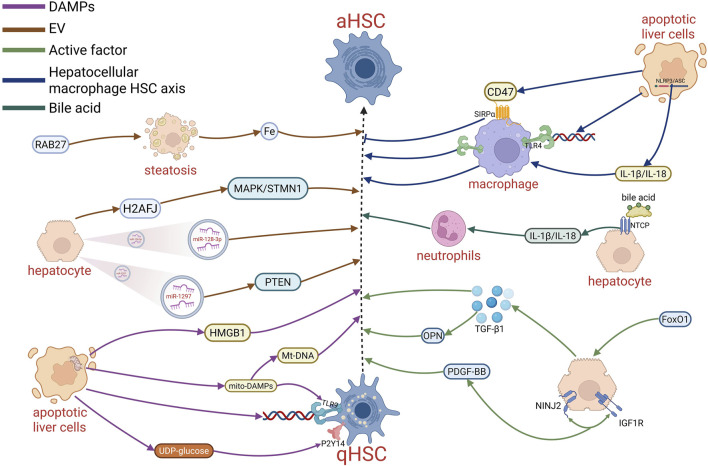
Communication between hepatocytes and HSCs.

DNA released from hepatocyte apoptotic body can interact with Toll-like receptor (TLR) 9 in HSCs to promote HSCs activation (Watanabe et al., 2007). High Mobility Group Box-1 (HMGB1) and DAMP released from necrotic hepatocytes, activates HSCs by regulating autophagy of HSCs (Li et al., 2018). In hepatocellular carcinoma (HCC), elevated HMGB1 released from gluconeogenic enzyme fructose 1,6-bisphosphatase (FBP1)-deficient hepatocytes triggers HSCs activation. Subsequently HSCs exhibit a senescence-associated secretory phenotype as a result of high levels of senescence-promoting CCN1, which contributes to HCC progression (Li et al., 2020) (Figure 2).

Nucleotide-binding oligomerization domain (NLRP3), a downstream effector of DAMPs in the inflammasome. In mice with mutant NLRP3, the hepatocyte cellular pyroptosis and HSCs activation were observed (Tsuchida and Friedman, 2017). In several liver injury models, injured hepatocytes are capable of releasing P2Y14 ligands, such as UDP-glucose and UDP-galactose, which activate the ERK and YAP signaling pathways mediated by the P2Y14 receptor to induce HSCs activation. This interaction lays the foundation for the pro-fibrotic DAMP-DAMP system (Mederacke et al., 2022). Mitochondria of damaged hepatocytes releases mito-DAMPs (mitochondria-DAMPs) which facilitate nonalcoholic steatohepatitis (NASH) inflammation via TLR9, and eventually exacerbate ischemia-reperfusion injury (Garcia-Martinez et al., 2016). Mt-DNA, the bioactive component of mito-DAMPs, activates HSCs released by hepatocytes to promote liver fibrosis. Mito-DAMPs are able to enter the circulation from the local microenvironment and are expected to serve as a biomarker of non-alcoholic fatty liver disease (NAFLD) in humans (An et al., 2020) (Figure 2).

Extracellular vesicles (EV) play an important role in hepatocyte and HSCs communication. It has been demonstrated that toxic lipid overload leads to the release of miR-128-3p-containing EV from hepatocytes. Upon internalization by HSCs, miR-128-3p inhibits PPAP-γ expression and promotes HSCs activation (Povero et al., 2015). Similarly, lipotoxic hepatocyte-derived exosomal miR-1297 promotes HSCs activation through PTEN pathway (Luo et al., 2021). EVs responsible for iron secretion from hepatocytes cause hepatocytes iron deficiency, hepatocytes iron deficiency and HSCs iron overload are features of NAFLD and NASH (Britton et al., 2018). Iron deficiency in hepatocytes facilitate the HIF-2α-ATF4 signaling pathway to promote lipogenesis. RAB27, a small GTPase critical for exosomes secretion, was increased in human NASH/NAFLD. Due to the reduction of Kupffer cells (KCs) in NASH, iron-containing EVs are phagocytosed by HSCs, resulting in HSCs iron overload, which enhances the accumulation of ROS in HSCs and promotes HSCs activation (Gao et al., 2022). ARRB1 (β-arrestin 1), a scaffolding protein in the intracellular signaling network, is involved in the regulation of autophagic flux in HCC (Lei et al., 2021). ARRB1 promotes liver fibrosis by hampering the autophagic lysosomal/multivesicular body pathway by activating Rab27a through p38 MAPK/ATF2 signaling (Liu X. et al., 2023). In addition, hepatocyte-derived EVs can deliver histone 2A family member J (H2AFJ) to HSCs, promoting HSCs activation through the MAPK/STMN1 axis (Liu B. et al., 2023) (Figure 2).

Besides DAMPs and EVs, bioactive molecules are another factors secreted by hepatocytes to regulate HSCs activity. NINJ2 encodes the cell adhesion molecule, nerve injury-inducible protein 2 (Ninjurin2), which modulates vascular endothelial cell activation and inflammatory responses, affecting atherosclerosis and coronary artery disease (Wang J. et al., 2017). In a model of MCD-induced liver fibrosis, NINJ2 knockdown attenuated liver fibrosis. Consistently, hepatocyte-specific NINJ2 overexpression was able to interact with IGF1R in hepatocytes, regulate PDGF-BB expression via the IGF1R-PI3K-AKT-EGR1 pathway, and promote HSCs activation via a paracrine manner, thus accelerating fibrosis in mice (Wang Y. et al., 2023). Forkhead box O protein 1(FoxO1), is a vital factor that participates in the insulin-PI3K-AKT signaling pathway in hepatocytes and regulates cell growth and metabolism (Barthel et al., 2005). FoxO1 was upregulated in a CCl4-induced mouse model of hepatic fibrosis, and knockdown of FoxO1 resulted in significantly reduced TGF-β1 secretion by hepatocytes, which impeded HSCs activation induced by paracrine TGF-β1 (Pan et al., 2024). OPN, a glycolipid protein highly expressed in the liver. E4-binding protein 4 (E4BP4), an important factor in NK cell development and immune regulation, has been reported to take part in the regulation of lipid metabolism (Zhao et al., 2021). In mice with hepatic fibrosis, TGF-β induced E4BP4 expression, which stabilized YAP independently of Hippo pathway, and promoted OPN expression, which caused HSCs activation via paracrine action. Consistent with the described results, the E4BP4 knockout model was reported to show alleviate hepatic fibrosis induced by diet in mice (Wang et al., 2024). Moreover, hepatocyte-secreted osteoblasts (Ma et al., 2023), IL-33 (Yu et al., 2024), and Monocyte-chemoattractant protein-induced protein 1 (Pydyn et al., 2024) also participate in the crosstalk between hepatocytes and HSCs in a similar manner (Figure 2).

Hepatocyte-macrophage-HSCs axis was reported to contribute to HSCs crosstalk. In NASH, macrophages connect hepatocytes with HSCs activation through exocytosis. In liver injury, apoptotic hepatocytes release soluble “find me” signals (ATP, S1P, and CX3CL1) to recruit macrophages, which are subsequently triggered to phagocytose apoptotic cells by phosphatidylinositol on the apoptotic cell membrane (Meadows et al., 2019). In NASH, hepatocyte necrosis is of much significance. CD47, the “don’t eat me” signal, is upregulated in necrotic hepatocytes. At the same time, SIPRα, the receptor for CD47, is also upregulated on macrophages, accompanied by an inhibition of macrophage clearance of necrotic hepatocytes. *In vitro* experiments and proof-of-concept mouse models, both anti-CD47 and SIPRα measures were administered, and increased macrophage phagocytosis of necrotic hepatocytes was shown, concomitant with mitigated liver fibrosis *in vivo*. These results suggest that blocking the CD47-SIRPα axis might be an effective strategy for the NASH (Shi et al., 2022). Gentiopicroside has been reported to inhibit the TLR4 and NLRP3 signaling pathways in macrophages, which brought the release of inflammatory factors, improvement of hepatocyte pyroptosis, and alleviation of the hepatic fibrosis (Yong et al., 2024). In a thioacetamide-treated mouse model, the expression of liver fibrosis markers and inflammatory responses mediated by macrophages was suppressed after the inhibition of HMGB1, TLR4 and the assembly of NLRP3/ASC inflammasomes induced by LPS/ATP in mouse peritoneal macrophages (Yao et al., 2024).

In addition, bile acids are also involved in the crosstalk between hepatocytes and HSCs. Sodium+/taurocholate cotransporting polypeptide (NTCP), a multichannel membrane transporter of the hepatocyte within the hepatic sinusoids, is responsible for nearly 90% of the bile acid transportation (Slijepcevic and van de Graaf, 2017; Jani et al., 2018). In bile duct ligation models, bile acids enter hepatocytes via NTCP to induce the expression of cytokines, such as IL-1β, CCL2 and CXCL2 (Cai et al., 2017), enhance neutrophil chemotaxis, and trigger an inflammatory response to initiate liver injury (L et al., 2017). For example, during cholestasis, elevated bile acid concentration can activate MAPK signaling in hepatocytes, which upregulates Egr-1, and thus stimulates neutrophil accumulation and expression of inflammatory cytokines in the liver (Allen et al., 2010). Actually, the function of inflammatory cytokines cannot be ignored in regard of HSCs activities. Moreover, some studies have shown that NTCP is also present on HSCs in patients with liver fibrosis, and the expression level is linearly correlated with the degree of fibrosis. Experimental results showed that NTCP can mediate the uptake of bile acids by HSCs, and in both vivo and vitro experiments, inhibition of NTCP was able to modulate HSCs inactivation (Salhab et al., 2022). Taken all together, bile acids may be able to serve as an important bridge linking hepatocytes and HSCs (Figure 2).

### 2.2 Interaction of HSCs with hepatocytes

Communication between hepatocytes and HSCs is reciprocal. There are limited studies on the role of exosomes from HSCs in hepatocyte regulation. In TGF-β1-activated LX-2, there is an increase in LX-2-derived exosomes containing miR-199a-5p, which are able to inhibit the proliferation of hepatocyte THLE-2 and promoting its epithelial mesenchymal transition (EMT) and senescence in an autocrine and paracrine manner (Lu et al., 2023). Constantly, it has been substantiated that HSC-derived miRNA-99a-5p in exosomes can suppress hepatocyte proliferation, promote hepatocyte apoptosis, and modulate hepatic fibrosis by targeting BMPR2 (Li F. et al., 2023). In young mouse hepatectomy models, matricellular protein CNN1 was rapidly elevated and induced HSCs senescence (Kim et al., 2013), which promoted HSCs to secrete IL-6 and CXCR2 ligands. IL-6 could activate STAT3 pathway, which induced the activation of the YAP pathway and synergistically activates ERK1/2 with CXCL2 to stimulate hepatocytes proliferation (Cheng et al., 2022). Neurotrophin-3, a profibrotic cytokine derived from HSCs (Wang S. et al., 2023), was reported to induce hepatocyte proliferation by stabilizing CCND1 through activation of the tropomyosin-related kinase B (TRKB) in hepatocytes (Trinh et al., 2023). In the knockdown model of C-type lectin-like transmembrane protein endosialin (EN), which presented on activated HSCs, the expression level of IGF2 was significantly upregulated, accorded with augmented hepatocyte proliferation. The negative effects on hepatocyte proliferation of EN is confirmed (Mogler et al., 2015), but more experimental evidence is required to substantiate the EN-IGF2 axis.

Type 2 diabetes mellitus (T2DM) has a bidirectional and close association with NAFLD/NASH (Younossi et al., 2019; Birkenfeld and Shulman, 2014), and the interaction between hepatocytes and HSCs carries a lot weight. Obesity, insulin resistance (IR), and lipotoxicity dynamically interact, leading to fat accumulation in hepatocytes and resulting in simple lipid storage. Furthermore, the activation and infiltration of hepatic macrophages, dendritic cells, and HSCs induce liver inflammation, thereby promoting the development of liver fibrosis (Mazzoccoli et al., 2018). Additionally, studies have shown that fat overload may render hepatocytes more susceptible to bile acid-induced inflammatory responses and apoptosis (Pusl et al., 2008), while bile acids in the liver may be served as an important bridge linking hepatocytes and HSCs to in the development process of liver fibrosis.

## 3 Cholangiocyte

In co-culture system, intrahepatic cholangiocytes, proximal intrahepatic cholangiocytes and HSCs all alter gene expression profiles mutually, with enhanced proliferation of intrahepatic cholangiocytes and activation of HSCs through the WNT pathway (Haaker et al., 2025). Although the exact explanation about the interaction between cholangiocytes and HSCs remains unclear, gal, histamine and leptin regulated cholangiocyte as well as HSCs proliferation and activation during cholangitis and fibrosis in MDR2 knockout mice were reported (Petrescu et al., 2020; Jones et al., 2016; Petrescu et al., 2022).

In NASH patients and mice with hepatic fibrosis, it was demonstrated that 12α-OH bile acids significantly activate HSCs via p38MAPK and ERK1/2 signaling pathways mediated by TGR5 (Xie et al., 2021). Transmembrane sphingosine-1-phosphate receptor 2 (S1PR2) plays an important role in cholestasis mediated by bile acids and inflammatory cytokines *in vivo* (Islam et al., 2024). In the context of cholestasis, it has been shown that taurocholic acid spurs HSCs activation through the S1PR2/p38MAPK/YAP signaling pathway (Yang J. et al., 2023). Previous research has confirmed that taurocholic acid was able to initiate the downstream ERK1/2 and AKT pathways via S1PR2 to facilitate cholangiocyte proliferation (Wang et al., 2017b). Similarly, investigations shown that conjugated bile acids could boost the development of cholangiocellular carcinoma via S1PR2 (Liu et al., 2014). Results above suggest a relationship linked by bile acids between HSCs and cholangiocytes. Besides, in the model of cholestasis, bile acids might be the key factor capable of connecting hepatocytes, HSCs, and cholangiocytes. Moreover, mast cells might mediate the interaction between cholangiocytes and HSCs.

It’s noted that cholangiocytes can express FXRβ and ASBT, imposing the influence of UDCA on the degranulation process in mast cell, in turn affecting HSCs activation (Meng et al., 2018).

The inflammatory cytokine, monocyte chemotactic protein-1 (MCP-1)/CCL2 is a monocyte chemokine released by blood cells. Activated cholangiocytes are able to release MCP-1 to induce the transformation of portal fibroblasts into myofibroblasts and promote their proliferation (Kruglov et al., 2006), as well as to promote the transformation of HSCs (Mühlbauer et al., 2003).

It has been shown that Apelin, the endogenous ligand for the G protein-coupled receptor, is a protein encoded by the apln gene (Tatemoto et al., 1998). The Apelin-APJ axis is involved in renal, myocardial, and renal fibrosis (Lv et al., 2017; Huang et al., 2016). It was demonstrated that in BDL-induced cholestatic liver fibrosis, Apelin-APJ induced cholangiocytes proliferation through Nox4/ROS/ERK signaling, as well as the increase of Col1α1, fibronectin1 (FN1), and TGF-β1 in cholangiocytes, while promoting the proliferation of vascular smooth muscle cells. HSCs activation was also triggered by Apelin-APJ via ROS in paracrine manner during cholestasis (Chen et al., 2021). Meanwhile, study revealed that Notch2, upregulated in the YAP pathway, aids the transformation of hepatocytes into cholangiocytes (Yimlamai et al., 2014). Furthermore, in hepatectomy model in young mice, the biliary tree fails to grow. In the presence of cholestasis, CCN1 spurs the proliferation of cholangiocytes by interacting with integrin αvβ3/αvβ5, inducing the expression of Jag1 via NF-κB, and further activating the NOTCH1 pathway (Kim et al., 2015). Taking the fact mentioned before that CNN1 can induce HSCs senescence into account, CNN1 might be another link between cholangiocytes and HSCs.

In cholestasis model, lncH19 in exosomes from cholangiocytes is preferentially taken up by HSCs rather than hepatocytes, which promotes G1/S conversion, enhances HSCs activation, proliferation, and thus promotes liver fibrosis (Liu R. et al., 2019). Experiment shows that miR-21 upregulation hampers HSCs apoptosis and aggravates hepatic fibrosis in alcoholic liver disease model (Francis et al., 2014). Furthermore, during cholestasis cholangiocytes may secrete exosomes containing miR-21, and therefore increase HSCs activation (Kennedy et al., 2016).

TGF-β/Smad pathway is a classical pathway in HSCs activation, and in cholangiocytes it can elevate the expression of HSC-activating genes. In terms of epigenetic modifications, KAT2A, an acetyltransferase in cholangiocytes, acetylates H3K9 and therefore trigger TGF-β downstream signaling pathways. These signaling pathways increase the expression of genes that assist HSCs activation, such as FN1 and SERPINE1. TGF-β signaling can specifically recruit KAT2A to the promoter regions of these genes, enhancing gene expression through H3K9ac. These gene products are ultimately released by cholanggiocytes in a paracrine manner, promoting HSCs activation as well as the progression of biliary fibrosis (Aseem et al., 2021). Enhancer of zeste homologue 2 (EZH2), an epigenetic regulator involved in the TGF-β pathway in cholangiocytes can directly target and inhibit FN1 in the cholangiocytes, which is an important paracrine factor in regulation of HSCs activation. EZH2 degradation leads to the upregulation of FN1 expression (Jalan-Sakrikar et al., 2019). Above all, these researches indicate the critical role of TGF-β in the communication between HSCs and cholangiocytes.

Crosstalk between HSCs and cholangiocytes is not restricted to themslves. During chronic cholestasis, myofibroblasts HSCs release soluble Hh ligands, which stimulate the expression of chemokine Cxcl16 in cholangiocytes, recruiting monocytes with chemokine cognate receptors, such as NK cells, and orchestrate the repair-related mechanisms of liver inflammation (Omene et al., 2009).

## 4 Dynamic regulation of HSCs and immune cells

**FIGURE 3:**
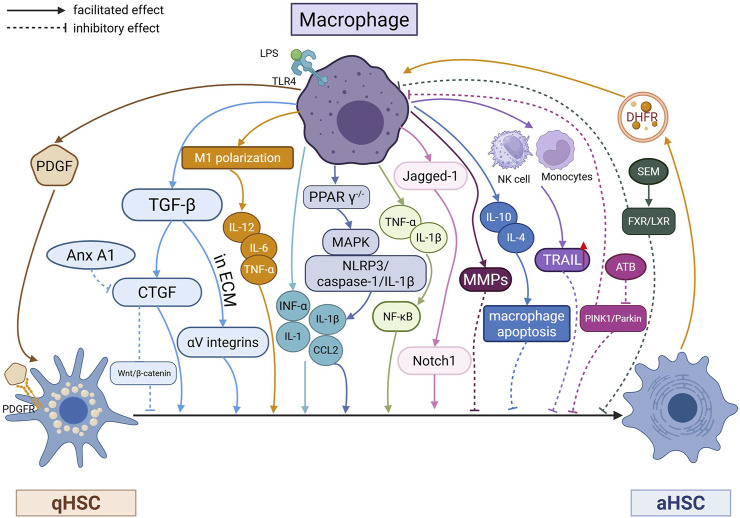
Hepatic macrophages and HSCs interactions and signaling pathways involved in HSC activation.

**FIGURE 4:**
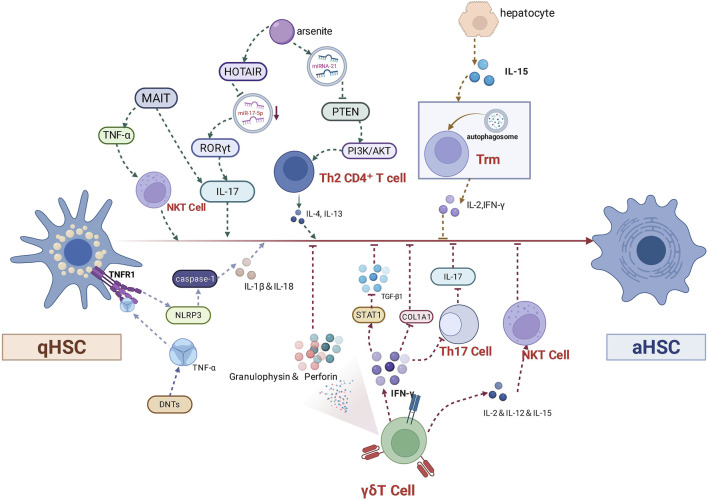
Multicellular immunomodulatory network in the process of HSC activation.

In addition to the HSCs, immune cells including macrophages (Figure 3), NK cells, T lymphocytes, B lymphocytes also play an important role, which can communicate with HSCs to promote or postpone the progression of liver fibrosis (Figure 4).

### 4.1 Macrophages/kupffer cells

#### 4.1.1 Hepatic macrophages

Hepatic macrophages can be classified into two groups based on their origin. One group is endogenous resident hepatic macrophages originating from KCs, which make up about 35% of the total number of nonparenchymal hepatic cells. KCs are capable of sensing tissue damage, clearing intestinal pathogens and damaged erythrocytes, and regulating iron and lipid metabolism (Scott and Guilliams, 2018). The other group is bone marrow-derived macrophage (BMDM), recruited from the circulation system, which account for only 5%–30% of the total number of hepatic macrophages. Under homeostatic conditions in the liver, embryonic-derived self-renewing tissue-resident KC tissue predominates. However, when hepatic tissue is damaged, “intravascular sentinel” KC cells are activated in rapid response to extrahepatic perturbations, expressing cytokines and signalling molecules. KCs are exposed to various substances via the portal circulation, and through pattern recognition receptors (PRRs) KCs can sense and remove pathogens (Scott and Guilliams, 2018). PRRs contain at least two families of sensing proteins: the TLRs and the NLRs, including PAMPs and alarmins. The TLRs can recognize bacterial products derived from the intestinal microbiota, such as LPS and peptidoglycan. Chemokines like CCL2, CCL5, CXCL9, CXCL10 and others, as well as their receptors CCD2 (and CCR5) recruit macrophages in circulation into the liver as infiltrating macrophages, which are involved in the inflammatory response and subsequent hepatic fibrosis process (Chazaud, 2014).

BMDM can be transformed into a Ly6c^lo^ population responsible for tissue remodelling (Ramachandran et al., 2012), with increased expression of MMP, growth factor and phagocytosis-related genes such as MMP9, MMP12, IGF1 and the transmembrane glycoprotein NMB (Gpnmb). BMDM-derived KCs (MoKCs) predominate in the hepatic KCs pool under cholestatic and toxic conditions (Li W. et al., 2023), and show enhanced proliferative and anti-apoptotic properties, while promoting repair and attenuating fibrosis. It is noteworthy that both KCs and BMDM highly express TGF-β, suggesting that both cells contribute to liver fibrosis.

Macrophages consist of heterogeneous subpopulations that exhibit different cellular functions. The current classical paradigm is the M1, M2 subpopulations, which includes classically activated macrophages (M1) and selectively activated (M2) macrophages derived from quiescent M0 macrophages that have undergo diverse differentiation pathways in different microenvironments. M1 macrophages, displaying a pro-inflammatory and anti-tumour phenotype, can present antigens, express cell surface markers such as CD80, CD86, TLR2, TLR4, and secrete pro-inflammatory cytokines, such as TNF-α, IL-6, IL-12, and other factors such as IL-1β, CCL2 and ROS (Shapouri-Moghaddam et al., 2018). In contrast, M2 macrophages display anti-inflammatory and pro-tumour phenotype, expressing arginase 1 and the cell marker CD206. M2 macrophages are capable of secreting anti-inflammatory cytokines such as IL-10, TGF-β, IL-4, and IL-13, and synergistically exerting anti-fibrotic effects (Du et al., 2016). The M2 macrophages can be subdivided into four subtypes: M2a, M2b, M2c, and M2d, where M2a and M2b macrophages mainly promote cell response and immunomodulation of Th2. M2c macrophages can inhibit the immune response and promote tissue remodelling. M2d macrophages mainly promote angiogenesis and tumour progression (Wermuth and Jimenez, 2015). Macrophage polarization is the tendency to convert into a specific functional state in the face of different stimulatory signals in different microenvironments. Inhibition of M1 macrophage polarization and promotion of M2 macrophage polarization are beneficial to the alleviation of liver fibrosis.

However, it is noted that a clear boundary between pro-inflammatory M1 macrophages and anti-inflammatory M2 macrophages has not yet been identified. During the pathogenesis process of endometriosis, ectopic endometrium-derived lactate induces polarisation of M2-type macrophages while inhibiting M1-type macrophages (Gou et al., 2023). Besides, M1-type BMDM have shown enhanced therapeutic efficacy in experimental liver fibrosis by modulating the hepatic microenvironment to recruit and modify endogenous macrophages and to regulate NK cell activation (Ma et al., 2017). Conversely, more studies support the existence of novel phenotypic transformation pathways of macrophages *in vivo* following specific alterations in the tissue microenvironment (Orecchioni et al., 2019).

Single-cell gene sequencing and spatial mapping were used to determine the presence of and isolate a scar-associated TREM2+CD9+ subpopulation of macrophages in the fibrotic ecotone of human cirrhosis. This subpopulation specifically expresses TREM2 and CD9, termed scar-associated macrophages (SAMΦ), and is derived from recruited and differentiated circulating monocyte. It is conserved among species, displays a pro-fibrotic phenotype and expands early in the progression of liver disease (Ramachandran et al., 2019). In contrast, monocyte-derived macrophages (MdM) infiltrating in metabolic dysfunction-associated steatohepatitis (MASH) are heterogeneous, which involves lipid-associated macrophage (LAM) expressing TREM2, Gpnmb, OPN1, CD63 and CD9 (Daemen et al., 2021), one assemble into macrophage aggregates around dying hepatocytes, forming hepatic corona-like structures (hCLS), which localize in areas filled with HSCs to promote fibrosis. In a model of high-fat diet-induced steatosis, specific fibrotic macrophage subpopulations, Ceacaml Msr1 Ly6C+++F4/80 Macl monocytes derived from Ly6C FcεRI-+ granulocyte/macrophage progenitors have been identified, which owns granulocyte characteristics, and was termed isolated atypical monocytes containing a nucleus, which is nucleus-containing atypical monocytes (SatM) (Satoh et al., 2017). Studies have identified NASH-associated macrophages (NAMs) with expression of triggering receptor 2 (Trem2), which is abundant on myeloid cells, by applying single-cell sequence on liver mesenchymal cells in NASH (Xiong et al., 2019). NAMs showed abundant expression of Gpnmb and CD9, which are associated with liver injury and fibrosis.

It is important to note that the relationship between inflammation and fibrosis is by no means “black and white.” Liver fibrosis is a dynamic process, with inflammation and fibrosis being two progressive stages. The inflammatory process not only initiates and maintains fibrosis, but also promote fibrinolysis and fibrosis resolution (Kisseleva and Brenner, 2021). Therefore, it is clear that purely anti-inflammatory and pro-inflammatory effects do not fit well with the different phases of the liver fibrosis. Liver inflammation precedes fibrosis in most liver diseases. And in a range of rodent models, liver fibrosis also induces inflammation.

#### 4.1.2 Hepatic macrophage-HSCs interactions

The activation of HSCs is the central event in liver fibrosis, in which HSCs transdifferentiate into myofibroblasts, losing vitamin A, up-regulating αSMA, and beginning to produce inhibitors of collagen and matrix degradation, leading to excessive ECM deposition. Current research suggests that the activation of HSCs is caused by the inflammatory activity of hepatic immune cells, especially macrophages. Macrophage-HSCs interactions play a key role in the process of liver fibrosis, and exhaustion or blockade of macrophage infiltration reduces HSCs activation and fibrosis (Matsuda et al., 2018). Notably, macrophages impose dual-acting effects on the process and regression of liver fibrogenesis. HSCs are bi-directionally regulated by macrophages. HSCs can be activated by a variety of signals, including cell-cell contacts with macrophages, cytokines derived from activated immune cells, and PAMPs or DAMPs (Tsuchida and Friedman, 2017).

##### 4.1.2.1 Hepatic fibrogenic stage

During the generative phase of liver fibrosis, macrophages produce various mediators that can activate HSCs. KCs, induced by PRRs, NLRP3 in inflammatory vesicles, release various cytokines which trigger the classical TGF-β and PDGF pathways to promote the interaction between HSCs and KCs, further activating HSCs and facilitating the process of liver fibrosis. For example, when binding to LPS, TLR4 induces the production of TNF-α, IL-1, IL-1β and CCL2 to activate myofibroblasts (Wei et al., 2019).

Macrophage-derived TGF-β is thought to be the most potent fibrotic agonist. IL-17 and IL-22 produced by neutrophils and helper T cells 17 (Th 17) can sensitise HSCs to TGF-β stimulation by up-regulating TGF-β receptor II (TGF-βRII), which in turn binds TGF-β on the surface of HSCs, inducing transdifferentiation and activation of HSCs. In turn, activated HSCs produce TGF-β to sustain activation through the feed-forward loop (Hammerich and Tacke, 2023). TGF-β can be deposited in the ECM (‘latent TGF-β’) and activated later by αV integrin-mediated HSCs contraction. CTGF, as a TGF-β downstream factor, mediates the increase of ECM synthesis and secretion (Cai et al., 2018; Trampuž et al., 2023). The main source of CTGF is HSCs, and blocking CTGF significantly inhibits the activation and proliferation of HSCs (Fan et al., 2023). TGF-β has also been found to induce autophagy in HSCs (Zhang J. et al., 2021), and provide energy and nutrients for HSCs activation. Hepatic macrophages, including KC- and BMDM-derived TNF-α and IL-1β, also enhance liver fibrosis by promoting aHSCs survival in an NF-κB-dependent manner (Pradere et al., 2013). But it appears to have no effect on HSCs activation (Pradere et al., 2013). PDGF secreted by endothelial cells and KCs binds to its receptor PDGFR and induces dimerisation and autophosphorylation of its subunits, causing constant activation and proliferation of HSCs (Mortezaee, 2018) (Figure 3).

There is bidirectional regulation and “reciprocity” between HSCs and macrophages. HSCs and macrophages both expressing the nuclear receptor peroxisome proliferator-activated receptor (PPAR), which can lead to a reduction in the activation of both cell types through PPAR γ agonism in HSCs and parallel PPAR δ agonism in macrophages in a mouse model of NASH (Ni et al., 2022) as well as in a clinical trial of 274 NASH patients (Francque et al., 2021; Lefere et al., 2020). In contrast, PPARγ-deficient macrophages exhibit chemotactic, pro-inflammatory and pro-fibrotic features, undergo anti-inflammatory polarisation, and promote IL-1β and CCL2-mediated migration and activation of HSCs associated with the MAPK and NLRP3/caspase-1/IL-1β signalling pathways (Ni et al., 2022). Subsequent administration of the pan-PPAR agonist lanifibranor can significantly ameliorate hepatic fibrosis (Francque et al., 2021). Jagged-1 expressed on liver macrophages and HSCs can interact with Notch1 on HSCs to promote Notch1-mediated HSCs activation and fibrosis (Yang et al., 2019). aHSCs contribute to macrophage migration and activation (Chang et al., 2013), then administration of the pan-PPAR agonist lanifibranor significantly improves liver fibrosis (Francque et al., 2021). aHSCs can assist macrophage phenotypic change to pro-inflammatory and pro-fibrogenic, while p38 inhibition in HSCs abrogates this change (Chang et al., 2013). Large amounts of the non-protein glycosaminoglycan HA are deposited in the ECM in hepatic fibrosis, which can regulate cellular functions via CD44 and TLR4 expressed in both HSCs and macrophages. HSCs are major source of CSF1, which plays a key role in inducing monocyte differentiation and proliferation within the ecotope after loss/exhuastion of KCs, suggesting that there is a reciprocal cycle of interaction between HSCs and KCs (Bonnardel et al., 2019) (Figure 3).

At the same time, PPAR also plays a key role in the development and treatment of T2DM-related NAFLD and is one of the key targets for intervention. PPARs regulate lipid and glucose metabolism, taking an important part in hepatic energy homeostasis and regulation of lipid synthesis. For example, PPAR-α primarily negatively regulates hepatic lipid uptake, PPAR-γ induces insulin sensitization and enhances glucose metabolism, and PPAR-δ regulates metabolism in the liver and other peripheral tissues, which reduces IR (Boyer-Diaz et al., 2020; Bojic and Huff, 2013). Intervention aiming at the PPARs can also alleviate NAFLD. For example, PPAR-α activators, such as certain fibrate compounds can inhibit NF-κB-induced inflammatory genes (Choudhary et al., 2019; Francque et al., 2015). Another example is the antidiabetic drug, pioglitazone, a PPARγ receptor agonist, which can activate the expression of M2-like anti-inflammatory genes in macrophages and restore the M1/M2 polarization (Luo et al., 2017).

Compared to single receptor activation, dual or multi-receptor PPARs activation is more effective, which can cause less side effects, and may even produce opposite effects to single-agent therapy. The dual PPAR-α/δagonist elafibranor (GFT505) has demonstrated efficacy in preclinical models of NAFLD, NASH, and liver fibrosis (Staels et al., 2013). Another case is the activation of PPAR-β/δ, which can reduce the expression of TNF-α or IFN-γ in KCs, thereby inhibiting inflammation. However, PPAR-β activation by L165041 promotes HSCs proliferation in both acute and chronic liver inflammation (Hellemans et al., 2003).

##### 4.1.2.2 Regression stage of liver fibrosis

During the regression phase of liver fibrosis, when underlying liver injury is removed and inflammation subsides, aHSCs can return to a quiescent phenotype, be inactivated, or be removed by apoptosis. Macrophages are a key driver of hepatitis and fibrosis, but they can also adopt a restorative phenotype to support tissue regeneration.

Neutrophils or phagocytes can stimulate macrophage phenotypic switching, leading to increased secretion of anti-fibrotic mediators MMPs, which promotes matrix degradation and regression of liver fibrosis (Popov et al., 2010). Pharmacological inhibition of pro-inflammatory monocyte recruitment using CCL 2 inhibitors in a mouse model of hepatic fibrosis was shown to shift the balance of intrahepatic macrophages towards a restorative phenotype, thereby accelerating fibrotic regression (Baeck et al., 2014). Macrophages in recovering stage also produce anti-inflammatory mediators such as IL-10, IL-4, induce apoptosis of pro-inflammatory macrophages and thus suppress fibrosis (Du et al., 2016). However, it is worth noting that this may only represent an active phenotypic transition and not in a separate subpopulation (Dal-Secco et al., 2015).

MdM, which includes LAM in MASH, exhibits the ability to degrade collagen in the hepatic crown-like structures (hCLS) formed in areas of hepatic fibrosis. HSCs and macrophages are in close contact in the hCLS and can share cytoplasmic contents with adjacent macrophages. HSCs can promote LAM markers such as TREM2, Gpnmb, OPN1, CD63 and CD9 expression, and enhance collagen degradation in macrophages (Chan et al., 2024). In addition, macrophages can induce or activate monocytes or NK cells, upregulate the expression of TNF-related apoptosis-inducing ligand (TRAIL), and promote HSCs apoptosis and collagen degradation (Ma et al., 2017).

In summary, infiltrating monocytes/macrophages can have both pro-fibrotic and anti-fibrotic effects on HSCs in an environmentally dependent manner. The original pro-fibrotic effect turns inhibitory after macrophage exhaustion. But, to complicate matters, macrophage exhaustion promotes further progression of fibrosis when the injury ceases (Duffield et al., 2005). Macrophage exhaustion alone is not the “dividing line” for the initiation of its pro-/anti-fibrotic function.

Related to the above involved cytokines and pathways, a series of drugs have a role in inhibiting the progression of liver fibrosis. Exosomes carrying dihydrofolate reductase (DHFR) play an important role in this process. Exosomes secreted by activated HSCs can further promote M1 polarisation of macrophages, while DHFR silencing in HSCs can reduce Lx-2 activation and M1 polarisation, thereby attenuating the development of liver fibrosis *in vitro* and *in vivo* (Peng et al., 2022). Astragalus stellatus (ATB) inhibits HSCs activation, inflammation and EMT through PXR-mediated PINK1/Parkin signalling and modulates HSCs-macrophage crosstalk, thereby alleviating hepatic fibrosis (Dou et al., 2024). Sesamol (SEM), a natural lignan compound isolated from sesame, inhibits hepatic fibrosis by interfering with HSCs-macrophage crosstalk through FXR/LXR axis-mediated autophagy inhibition (Jiang et al., 2024) (Figure 3).

Besides, some modifications to macrophages can also interfere with their communications with HSCs. For example, modification of the chimeric antigen receptor (CAR) of BMDM to form CAR macrophages (CAR-MS) alters their phagocytosis of HSCs, recruits and presents antigens to T cells, and generates specific anti-fibrotic T cell responses to reduce fibroblasts and liver fibrosis in mice (Dai et al., 2024). Lipopolysaccharide modification of clodronate liposomes (CLD-lipos) can effectively exhaust macrophages to significantly increased macrophage clearance and apoptosis of HSCs (Zhang et al., 2024).

#### 4.1.3 The hepatocyte-macrophage-HSCs axis

More than 70% of patients with T2DM are accompanied by NAFLD, and their coexistence and interaction increase the stubbornness of NAFLD. T2DM can promote the progression of NAFLD to NASH via the hepatocyte-macrophage-HSCs axis. Obesity and IR contribute to the onset of T2DM, leading to significant events like lipid metabolism abnormalities, inflammation, and hepatocyte apoptosis (Su et al., 2018). Among these, lipid metabolism abnormalities result in increased lipolysis, fatty acid influxing into the liver, which foster abnormal fat accumulation in the liver, inducing oxidative stress to damage hepatocytes. KCs are activated after ingesting apoptotic fat-rich hepatocytes and free cholesterol, starting pro-inflammatory activation and releasing excessive inflammatory cytokines such as TNF-α, IL-1, and IL-6, facilitating the progression of NAFLD to NASH. Furthermore, KCs lead to activation of HSCs, enhancing liver fibrosis (Su et al., 2018).

Conversely, the inflammatory pathway can interfere with the insulin signaling pathway, leading to a vicious cycle involving obesity, IR, lipid metabolism abnormalities, inflammation, and NAFLD (Ballak et al., 2018). TNF-α can activate its receptor, promoting IRS1 phosphorylation and resulting in IR, which in turn leads to lipid metabolism disorder (Ishizuka et al., 2007). At the same time, TNF-α can also activate downstream inflammatory pathways through the NF-κB signaling pathway (Austin et al., 2008).

### 4.2 T-lymphocytes of NPCs that are not “NPCs”

The liver is a tolerant organ that releases DAMP, PAMP molecules that activate antigen-presenting cells (APCs) when it is stimulated by multifactorial factors such as bacterial and viral infections and hepatocellular damage. Many of the nonparenchymal cell populations therein act as APCs to present antigens and co-stimulatory molecules to ILTC. ILTC preferentially suppresses adaptive immunity by killing or inactivating T cells or by inducing the maturation of initial T cells into regulatory T cells, or say Tregs that suppress CD4 and CD8 T cell responses. Instead of initiating the activation of T cells, APCs prefer to induce T cell tolerance. APCs upregulates co-inhibiting ligands like programmed cell death-ligand 1(PD-L1) and programmed cell death-ligand 2(PD-L2) after IFN stimulation and produce immunomodulatory cytokines. When negative regulatory signalling in effector cell is defective, the immune system can be activated but pathogen defence fails. The presence of the suppressive pathway maintains immune homeostasis, with self-preserving T-cell exhaustion to prevent over-activation and exhaustion of immune cells, but meanwhile sustains appropriate clearance of pathogens and tumour cells. For example, in chronic hepatitis caused by Hepatitis B Virus (HBV), effector CD8^+^ T cells show a multilayered “exhaustion” phenotype, with a markedly reduced proliferative capacity and the production of IFN-γ, IL-2, TNF-α, granzymes, or perforin (Hoogeveen et al., 2019), which may be associated with the upregulation of co-inhibitory receptors like programmed death 1(PD-1), cytotoxic T lymphocyte-associated antigen 4 (CTLA) (Isogawa and Tanaka, 2015). The imbalance between effector T cells and Tregs, especially Th17/Tregs, therefore underlies the loss of autoantigenic immune tolerance and plays a key role in the progression of liver fibrosis.

ILTCs in general produce three types of cytokines: type 1 cytokines drive inflammation by initiating the migration of effector cells (Ruf et al., 2021). In contrast, type 2 cytokines such as IL-4 and IL-10 suppress immune-associated cytotoxicity through activation of Tregs or myeloid-derived suppressor cells (MDSC) (Shiri et al., 2024; Yang et al., 2017). Type 3 cytokines such as IL-17 and IL-22 can stimulate HSCs, which can lead to ECM deposition and fibrosis (Zhang et al., 2009).

HSCs also have strong immunomodulatory activities, including suppressing T cells through direct cell-to-cell contact mediated by PD-L1 on the HSCs surface (Charles et al., 2013), and enhancing suppression of T- and B-cell responses by promoting proliferation of myeloid-derived suppressor cells (Li et al., 2014). HSCs can inhibit Treg proliferation by activating latent TGF-β1 in hepatic HSC cells via glycoprotein-A repetitions predominant (GARP), a marker on the surface of activated Tregs (Li et al., 2015). NKT cells can exacerbate hepatic fibrosis by producing IL-4 that induces GARP expression on HSCs. Therefore, targeting ILTC to modulate their functions and interactions with HSCs is important for aiding the treatment of liver fibrosis by restoring immune homeostasis and reducing liver inflammation (Figure 4).

#### 4.2.1 IL-17A mediation

It is an important way that ILTC-associated IL-17A exacerbates fibrosis progression through activation of HSC/hepatic myofibroblasts (HMF). Studies found that MAIT cells isolated from PBMCs of patients with alcohol induced liver disease (AILD) regulated HSCs *in vitro* and induced a pro-fibrotic phenotype through IL-17 secretion (Böttcher et al., 2018). In a mouse model of arsenite-induced hepatic fibrosis, miRNA-21 disrupts the metabolic reprogramming of CD4 T cells through the PTEN/PI3K/AKT pathway to promote polarisation towards a Th2 phenotype, upregulating pro-fibrotic factors such as the transcription factors GATA binding protein 3 (GATA3), and pro-fibrotic factors like IL-4 and IL-13, thus promoting the activation of HSCs (Sun et al., 2022). Arsenite can downregulate miR-17-5p via HOTAIR, which in turn promotes the nuclear receptor retinoic acid receptor-associated orphan receptor γt (RORγt) and pro-inflammatory cytokine IL-17-mediated differentiation from CD4 T-cells to Th17 cells, which further activate the HSCs and facilitate fibrosis (Wu et al., 2023). Both HSCs and Th17 cells can secrete CCL20, a Th17 chemotactic agent, of which the corresponding receptor, CCR6, is expressed in Th17 and γδ T cells. Therefore, it is likely that CCL20 directly mediates HSCs and Th17 cell recruitment in acute alcoholic hepatitis and fibrosis (Annunziato et al., 2007). Researchers also found that HSCs could upregulate the number of Th17 cells and Tregs in liver tissues of patients with advanced HBV-associated hepatic fibrosis through the PGE2-/EP2 and EP4 pathways (Li et al., 2017). In patients with chronic liver disease, IL-33, the Th2 cell chemotactic agent and alarmin, enhances the recruitment and activation of CD4^+^ T cells with Th2-like properties, which activate HSCs in a paracrine IL-13-dependent manner and promote fibrosis (Reißing et al., 2024).

#### 4.2.2 Receptor mediation

Pattern recognition receptors expressed at high levels on parenchymal and non-parenchymal liver cells are mainly three, the TLR, NOD-like receptor (NLR) and RIG-like receptor (RLR). They recognize microbial components not found in mammalian systems and also play an important role in the interaction of T cells with HSCs to promote liver fibrosis. They recognize microbial components that are not found in mammalian systems and also play an important role in T cell-HSCs interactions to promote liver fibrosis. For example, HSCs can increase the immunosuppressive function of natural Foxp3+ Treg through indoleamine 2,3-dioxygenase (IDO)-induced activation of aryl hydrocarbon receptor (AhR) (Kumar et al., 2017). Self-noncoding RNA-containing exosomes from HSCs can mediate the activation of TLR3, which can further exacerbate hepatic fibrosis by enhancing the production of IL-17A by γδ T cells (Seo et al., 2016). Furthermore, TLR is also a member of the gut-liver axis, and toll-like receptor ligands derived from the intestine can directly affect Tregs function by binding to TLR on the Treg surface and inducing trans-differentiation into non-inhibitory Tregs (Osei-Bordom et al., 2020).

Blocking retinol metabolism is a promising therapeutic target and pathway to protect against T cell-induced hepatitis by increasing Tregs migration. HSCs are rich in retinol, containing 70% of the body’s retinol and play an important role in retinol homeostasis. Semaphorin-4D (Sema4D), which is involved in T-cell initiation and antibody production, is upregulated during HSCs activation. Its knockdown suppresses the homeostasis of Th1, Th2, Th17, and Treg cells through inhibition of the AOX1/retinoic acid receptor-α(RAR-α) pathway in retinol metabolism, which impedes the progression of hepatic fibrosis (Wang L. et al., 2023). HSCs are involved in Tregs expansion and differentiation through IFN-γ-mediated stimulation and Raldh1-derived RA production, respectively (Dunham et al., 2013). In the presence of the wide-spectrum ADH inhibitors 4-methylpyrazole (4-MP) and IFN-γ, HSCs increase the gene expression of CCR2 and IL-10 in Tregs. However, in the absence of Raldh1, HSCs can increase Tregs migration in a CCL2/CCR2-dependent manner, which ameliorates liver injury (Lee et al., 2015). C-AMP responsive element modulator-α (CREMα) is a central mediator of T-cell pathogenesis and contributes to the increase of IL-17 expression in patients with autoimmune diseases. Retinoic acid of HSCs origin induces the Treg phenotype in hepatic T-cells in transgenic mice overexpressing CREMα, leading to Tregs protective response (Kuttkat et al., 2017).

Double-negative T-cells (DNTs) are one of the important immune cells that mediate the progression of liver fibrosis, which can interact with HSCs through the NLRP3 receptor-mediated pathway. Studies found that the proportion of DNTs was increased in patients with liver fibrosis (Yang Y. et al., 2023). The transcription factor AP-4 (TFAP4) promotes the transcriptional expression of OX40, which in turn promotes the differentiation and survival of DNTs through its downstream genes such as NF-κB, Bcl-2, and surviving. And DNTs with high expression of TNF-α promote the activation of HSCs and exacerbate hepatic fibrosis through the TNFR1-NLRP3 (Han et al., 2023).

FasL-Fas is also an important pair of partner. Single-cell transcriptome analysis and fluorescence-activated cell sorter (FACS) analysis of NASH mouse models revealed that hepatic CD8 tissue-resident memory CD8 T (CD8 Trm) cells maintained by tissue IL-15 attracted HSCs in a CCR5-dependent manner and predisposed aHSCs to FasL-Fas-mediated apoptosis (Koda et al., 2021). When HSCs are co-cultured with γδ T cells, γδ T cells can eliminate aHSCs in co-culture through activation of natural cytotoxicity triggering receptor 1 (NKp46), TRAIL, and FasL mechanisms (Liu M. et al., 2019).

In addition, it should not be overlooked that the previously mentioned CTLA-4 family is also a T-cell surface receptor that competitively antagonises the T-cell co-stimulatory receptor CD28 and prevents T-cell activation. PD-1, a member of the CTLA-4 family, plays a similar role to play in maintaining homeostasis of effector T-cell function.

#### 4.2.3 T-cell autophagy

T-cell autophagy has also been recently found to be strongly associated with liver fibrosis. Human hepatic memory CD8+T (TRM) cells have different phenotypes, transcription factor expression, and the ability to maintain effective IL-2 and IFN-γ production in tolerant livers (Fernandez-Ruiz et al., 2016). Primary HSCs or prototypic hepatic secreting cytokine IL-15 induce autophagy in TRM cells in parallel with tissue homing/retention markers to adapt to mitochondrial depolarisation, optimise function and acquire tissue residency (Swadling et al., 2020). Three promising autophagy-associated differentially expressed genes as therapeutic biomarkers for hepatic fibrosis, including autophagy - related 5 (ATG5), retinoblastoma inducible coiled-coil protein 1 (RB1CC1) and PARK2, among which RB1CC1 may promote the progression of liver fibrosis by regulating macrophages, Th17 cells, NK cells and CD56dim natural killer cells (Huang et al., 2024).

#### 4.2.4 Apoptosis and lysis

γδ T cells are an interesting “sword” fighting against the process of liver fibrosis and like to cooperate with NK cells to communicate with HSCs to fight fibrosis. γδ T cells are divided into γδ T1 and γδ T17 functional subpopulations defined by IFN-γ and IL-17 production respectively. γδ T cells express γδ TCRs composed of Vδ and Vγ chains and the TCR pool is diversified through V (DJ) DNA recombination. Even though it can express TCRs it still has innate immune cell characteristics. They can also rapidly sense environmental changes through the expression of integrins, chemokines, and activate the relevant cells in a TCR-independent manner to rapidly generate effective effector responses. In most cases, γδ T cells, especially the γδT1 subpopulation, have a very potent cytotoxic effect on HSCs, by producing IFN-γ (Hammerich et al., 2014), or with the help of chronic hepatitis-promoted expression of NKp46 to directly kill aHSCs (Liu M. et al., 2019), which at the same time increases the cytotoxicity of NK cells against aHSCs to fight fibrosis. In addition, γδ T cells, especially the recently discovered Vγ4 γδ T subpopulation, can lead to adoptive transfer, which directly induces HSCs apoptosis and enhances NK cell-mediated HSCs cytolysis, thus significantly alleviating hepatic fibrosis (Liu M. et al., 2019). γδ T cells also promote the anti-fibrotic capacity of conventional natural killer (cNK) cells and liver resident NK (lrNK) cells while enhancing cytotoxicity against activated HSCs (Liu M. et al., 2019).

#### 4.2.5 Multicellular immunomodulatory network

Tregs can inhibit NK cell activation when co-cultured with HSCs in a cell-contact-dependent manner involving CTLA-4, which in turn inhibits NK cell killing of aHSCs and downregulates natural killer group 2, member D (NKG2D) ligands of NK cell receptor ligands activated on HSCs such as UL16-binding protein-liketranscript 1/UL16-binding protein 2(ULBP-1/2) (Langhans et al., 2015). In NASH livers enriched with C-X-C motif chemokine receptor 6(CXCR6) CD8 T cells with low activity of the forkhead box protein O1(FoxO1) transcription factor, cells can be auto-aggressively killed in an MHC class I non-dependent manner via signals from the P2X7 purinergic receptor. Meanwhile, IL-15 can induce FoxO1 downregulation and CXCR6 upregulation, which together make liver-resident CXCR6 CD8 T cells susceptible to metabolic stimuli, including acetate and extracellular ATP, and collectively trigger self-attack (Dudek et al., 2021). Hepatic macrophages accumulated in CCl4-induced liver injury can produce IL-1β to promote the activation of mTORC2 signalling in γδ T cells, which upregulates T-bet expression and ultimately promotes CXCR3 transcription to drive the migration of γδ T cells. Hepatic γδ T cells have reached destination and can then cause cytotoxicity to aHSCs in a FasL-dependent manner to ameliorate hepatic fibrosis. They can also secrete IFN-γ to inhibit the differentiation of pro-fibrotic Th17 cells (Liu et al., 2022). γδ T cells can promote the anti-fibrotic capacity of cNK cells and lrNK cells by enhancing cytotoxicity against aHSCs. For example, cNK cells can prevent hepatic fibrosis by TRAIL-dependent manner to kill aHSCs (Liu M. et al., 2019). CCR6 γδ T cells accumulate in fibrotic livers in the vicinity of HSCs, which limit hepatic fibrosis by producing IL-17 and IL-22 (Hammerich et al., 2014). In a mouse model of cholestatic liver fibrosis, activated MAIT cells can ameliorate fibrosis by direct cell-to-cell contact and NK cytotoxicity enhanced by TNF-α to HSCs (Jiang et al., 2022).

### 4.3 NK cells

Nearly half of the lymphocytes in the liver are NK cells, which have a relatively diverse cellular classification with the continuous development of various high-dimensional cellular heterogeneity analysis techniques such as spectral cytometry, time-of-flight cytometry (CyTOF) or scRNA-seq. They can usually be classified into transient cNK cells and lr-NK cells (Mikulak et al., 2019). They can also be further divided into three functional subsets, tolerogenic NK cells, regulatory NK cells and cytotoxic NK cells (Fu et al., 2014). CD11b+CD27- NK cells are likewise immature NKs with differentiation potential, displaying potent cytolytic function, as previously mentioned high cytotoxicity to HSCs (Fu et al., 2011).

Notably, NK cells play a dual role in liver fibrosis. They can promote hepatic fibrosis by producing pro-fibrotic cytokines such as IL-4 and IL-13 (Wehr et al., 2013), while they can also inhibit fibrosis by producing IFNγ to kill HSCs under specific conditions (Park et al., 2009). cNK cells can resist hepatic fibrosis by killing aHSCs in a TRAIL-dependent manner (Liu M. et al., 2019). Meanwhile, in a mouse model of hepatic fibrosis found that retinoid signalling can sensitise HSCs to NK cell killing by upregulating the NKG2D ligand RNA export 1 homolog (RAE1) (Radaeva et al., 2006). In humans, aHSCs can similarly lead to the upregulation of the NKG2D ligands ULBP-1/2 and MHC class I chain-related A/B (MIC-A/B), which can in turn trigger NK cell killing (Glässner et al., 2012).

In addition to the mentioned modulation of receptor-ligand to increase the sensitivity of NK-HSCs recognition to promote NK killing, interference with external factors such as CTLA-4-associated T cells, prostaglandin E receptor 3 (EP3), dihydromyricetin (DHM), microRNA, can help to enhance NK killing of HSCs to inhibit liver fibrosis. In chronic hepatitis liver fibrosis, when CTLA-4 regulatory cells are co-cultured with HSCs, they can secrete cytokines such as IL-8 and TGF-β1 to inhibit the expression of the NKG 2D ligands, MIC-A/B, as well as the expression of HLA class I on HSCs, and thus inhibit the activation of NK cells (Langhans et al., 2015). G-protein-coupled receptor (GPCR) EP3 promotes CD11b+ CD27^+^ NK cells Itga 4-VCAM 1-dependent cytotoxicity of HSCs (Glässner et al., 2012; Tao et al., 2022). PD-1/PD-L1, which balances the function of effector T cells as mentioned, also plays an important role in NK cells. With NK cells dysfunction at later stages of chronic hepatitis, PD-1 as a marker of NK cells exhaustion, is increased, especially in some hepatocellular carcinoma (Peng et al., 2022). DHM enhances IFN-γ expression through the NF-κB/STAT3 pathway to improve NK cell killing, inhibits HSCs activation and significantly improves the CCL4-induced liver fibrosis (Zhou et al., 2021). The expression of CCR2 and IL-10 in Tregs enhanced by HSCs can equivalently inhibit IFN-γ production in NK cells to promote aHSCs apoptosis and inhibit HSCs activation, attenuating liver fibrosis (Yi et al., 2014). In addition, EVs and exosomes often contain a variety of functional miRNAs, which carry out mail-type communication between cells. NK cells can hamper cytotoxic killing of HSCs with the help of miRNAs. miR-233 and miR-96-5p in NK cells exosomes could block autophagy of aHSCs by inhibiting the expression of autophagy-related 7 (ATG 7) (Wang L. et al., 2020; Yu et al., 2018).

However, it is worth noting that some studies have found that the anti-fibrotic activity of NK cells is negatively correlated with the progression of hepatic fibrosis in patients with chronic hepatitis C, which is most likely due to the fact that the large increase in the secretion of TGF-β1 by aHSCs can inhibit the degranulation of NK cells and IFN-γ production, and thus suppress their anti-fibrotic effects (Shi et al., 2017). Moreover, conversely, these new NK cells, instead of fighting HSCs, significantly promoted the proliferation of HSC-T6 or LX-2 cells co-cultured with them, and further increased the expression of collagen type I and α-SMA. Therefore, the duration of different liver injury conditions, in particular, is important for the direction of NK cell properties, rather than simply saying that NK cells maintain a single identity throughout the entire hepatic fibrosis process. So, the interaction between NK cells and HSCs is a promising approach to alleviate liver fibrosis.

Currently, CAR engineering of NK cells is gradually being proven to treat liver fibrosis and HCC with high specificity and few side effects. In the present, allogeneic induced pluripotent stem (iPS) cells were used to conduct CAR-NK cell therapy, a replacement cell for HSCs, and more than 1,340 clinical trials have been conducted by 2021, which is promising for the future (Arias et al., 2021).

### 4.4 B-lymphocytes

It has been reported that many B cell subpopulations have been identified, including B-1, B-2, and regulatory B cells. B-1 cells are mainly derived from fetal liver, and B-2 cells are derived from bone marrow (BM). Regulatory B cells (Bregs) can suppress the immune response mainly through the production of the anti-inflammatory cytokine IL-10 (Wang Y. et al., 2020). Recent evidence suggests that adaptive immune cells are an important regulatory factor in metabolic dysfunction-associated steatotic liver disease (MASLD). In liver biopsies from patients with MASLD, the accumulation of intrahepatic B cells was positively correlated with MASLD activity scores (Li and Xia, 2024). In addition, intrahepatic B cells may also be involved in MASLD by inducing the secretion of IL-6, TNF-α and IgG2a as well as enhancing the activation of CD4^+^ T cells and their differentiation to Th1 cells (Zhang et al., 2016). Aapelin/APLNR promotes the migration and activation of B cells, which results in the expression of cytokines such as AHNAK, COL6A3, IL10, IRF9 and RFX2, affecting liver fibrosis in MASLD (Jiang et al., 2025). Hepatic B-cell infiltration has been observed in experimental models of MASLD (Li and Xia, 2024). In advanced MASH in humans and mice, IL21 is activated through the IL-21R-STAT1-c-Jun/c-Fos-IgA regulatory pathway, leading to the induction of immunosuppressive IgA+ B cells and the accumulation of IgA-producing plasma cells, which inhibit anti-tumour cytotoxic CD8^+^ T cells through the expression of PD-L1 and IL-10, thus facilitating the emergence of HCC (Sutti and Albano, 2020; Xie et al., 2024). It has been shown that TLR4 ligands can activate B cells through the TLR4-MyD88 pathway, leading to NF-κB stimulation. Also, endogenous DNA-containing antigens released by dying cells can activate B cells via TLR9 (Barrow et al., 2021). In summary, through different pathways, B cells can be activated to produce different antibodies and cytokines, contributing to the development of MASLD and MASH, which may lead to the emergence of liver fibrosis (LF) and HCC.

In autoimmune hepatitis (AIH), autoantibody LKM-1 from B cells can target and recognize CYP2D6 on hepatocytes, which may be directly involved in autoimmune liver injury. Tetraspanin 1 (TSPAN1) B cells secrete a large number of inflammatory cytokines, which suggests their involvement in autoimmune hepatitis liver injury and its progression (Longhi et al., 2024). Corticosteroids, ursodeoxycholic acid, and rituximab have been reported as therapeutic agents for immune-mediated liver disease, where prednisolone and rituximab can act on B cells therapeutically (Cargill and Culver, 2021). B cells are also involved in liver fibrosis. Experiments using single-cell analysis, found that hepatic B cells in CCl4-induced LF were predominantly naïve B cells (more than 98%), which suggests that hepatic B cells function mainly as innate immune cells in the context of the present studies (Feng et al., 2025). In acute chronic liver failure (ACLF) livers resulting from further progression of hepatic fibrosis, the fraction of naive B cells was reduced, and CD27CD21 atypical memory B cells (atMBC) expressing higher CD11c and lower CD80 molecules were abundant. Besides, preferential accumulation of intrahepatic CD27CD38 plasma cells was shown. Expression of greater amounts of CD273 (PD-L2) and secretion of higher levels of granzyme B and IL-10 were observed. These all are positively correlated with disease severity indices (Zhao et al., 2022). As the disease progresses, patients with cirrhosis have lower levels of CD27 MBCs and higher levels of naïve B cells, and these B cells in patients with cirrhosis suffering from more advanced liver disease show a maturation transition towards CD27 MBCs, double-negative B cells and plasmablasts compared to patients with earlier stages of the disease (Cardoso et al., 2021). In this regard, treatment of fibrosis may prevent the disease from deteriorating again.

It has been experimentally shown that HSCs can have an effect on B cells. Using isolated mouse primary HSCs, we found *in vitro* and *in vivo* that HSCs directly inhibit B cells via PD-L1, which interacts with PD-1 on B cells to inhibit proliferation and antibody/cytokine production in activated B cells (Li et al., 2016). Retinoic acid produced by HSCs enhances B cell survival, plasma cell marker CD138 expression and IgG production. These activities were reversed when the retinoic acid inhibitor LE540 was administered. Meanwhile, transcriptional profiling in fibrotic hepatic B cells showed increased expression of genes related to NF-κB activation, pro-inflammatory cytokine production and CD40 signalling in activated B cells, suggesting that these B cells are activated and may act as inflammatory cells (Thapa et al., 2015). In turn, B cells can have an effect on HSCs. Surprisingly, B cells can selectively affect ECM production without affecting the number of α-SMA-positive myofibroblasts (Li et al., 2016). Experiments have shown that lymphocyte ablation (especially B cells) strongly inhibits HSCs activation and ECM deposition, and enhances the transition of HSCs to cellular senescence. Conversely, B cells maintain LF and foster the growth of HCC in chronic injury by modulating the innate components of inflammation, limiting the extent of HSCs’ senescence and promoting the pro-tumourigenic TNF-α/NF-κB pathway (Faggioli et al., 2018). This pro-fibrotic function of B cells can be attributed to the elicitation of IL-6 and the helper T 1 (Th1) response, which disrupts ECM renewal in chronic inflammation. Meanwhile, B cells can be pro-fibrotic by activating HSCs through the production of TGF-β1 and TNF-α or by inhibiting ECM degradation through TIMP-2. In addition, B-cell receptor (BCR) restriction reduced B-cell maturation, activation, and effector responses in the liver, accompanied by a reduction in T-cell and macrophage-mediated inflammation. BCR restriction attenuated hepatic fibrosis in mice, which was associated with a reduction in IgG production on HSCs and a decrease in the expression of the Fc-γ receptor (Barrow et al., 2023). What’s more, it has been shown that neuropeptide (NPY) is upregulated in human MASLD and that B cells may require dipeptidyl peptidase 4 (DPP4) to contribute to CCL4-induced hepatic fibrosis via NPY to induce maximal collagen production (Wang XM. et al., 2017).

In summary, we focus on the pro-inflammatory and pro-fibrotic roles of B-cells in the liver, which provides a new research direction for immunotherapy of liver diseases.

## 5 Interaction HSCs with other stromal cells

### 5.1 Liver sinusoidal endothelial cells (LSECs)

In acute liver injury, LESCs induce hepatocyte regeneration via CXCR7, whereas fibrosis is mediated by CXCR4 and fibroblast growth factor receptor 1 (FGFR1) in the setting of long-term injury (Ding et al., 2014). Zinc-finger E-box-binding homeobox2 (Zeb2), which is expressed in HSCs and regulates HSCs activation and apoptosis (Zhou et al., 2016; Yang et al., 2018). It was displayed that Zeb2 knockdown promoted LSECs capillarization and affected the expression of genes related to LSECs-HSCs communication. Genes that attenuate fibrosis such as GDF15 (growth/differentiation factor 15), LTF (lactoferrin), and IGF1 are downregulated. Changes in capillarization and gene expression simultaneously promote HSCs activation (de Haan et al., 2022).

In a healthy liver, VEGF mediates NO synthesis by nitric oxide synthase within LSECs to inhibit and reverse HSCs activation (Tsuchida and Friedman, 2017). During fibrosis, VEGF derived from hepatocytes and HSCs maintains the fenestration phenotype of LSECs through the eNOS-sGC pathway and non-NO-dependent pathways (DeLeve, 2015). In fact, capillarization of LSECs precedes the activation of HSCs and macrophages after liver injury (Poisson et al., 2017).

The Notch signaling pathway plays an essential role in maintaining hepatic sinusoidal and hepatocyte homeostasis, whereas the activation of the NOTCH pathway in LSECs inhibits the eNOS/sGC signaling pathway, resulting in the promotion of LSECs capillarization, impairment of liver regeneration, and hepatic fibrosis progression (Duan et al., 2018). Delta-like protein 4 (DLL4), a ligand of the Notch signaling pathway, is highly elevated in LSECs in liver fibrosis. It has been shown that DLL4 overexpression increases endothelin-1 (ET-1) synthesis and enhances HSCs coverage in the hepatic sinusoids (Chen et al., 2019). In the case of portal hypertension caused by cirrhosis, ET-1 was able to strengthen the HSCs contraction, leading to an increased vascular resistance. In the case of stress and chronic liver disease, however, ET-1 encourages HSCs differentiation into myofibroblasts (Kojima et al., 2001). Furthermore, in terms of epigenetic regulation, AIRN is an antisense lincRNA of the nuclear-localized IGF2R that enters the cytoplasm in a stable form after being spliced (Seidl et al., 2006). Kruppel-like factor 2 (KLF2), a transcription factor capable of positively regulating the eNOS-sGC pathway maintains LSECs differentiation, has been demonstrated to facilitate the inactivation of HSCs activation and apoptosis to alleviate liver fibrosis by Simvastatin (Marrone et al., 2015). It has been reported that AIRN negatively regulates HSCs activation by maintaining the normal LSECs phenotype through the KLF2-eNOS-sGC pathway. Meanwhile, AIRN in hepatocytes promotes hepatocyte proliferation via LSECs paracrine secretion of Wnt2a and HGF (Hepatocyte Growth Factor) (Chen et al., 2022) directly and indirectly. POFUT1 is an important regulator in Notch signaling, which can control angiogenesis in coronary artery endothelial cells during embryogenesis (Wang et al., 2017d). Experiments displayed that in the hepatic fibrosis mice model with POFUT1 knockout in LSECs, POFUT1 deficiency inhibited the KLF2-eNOS-sGC signaling axis, promoting injury-induced LSEC capillarization, while up-regulating the expression of fibrinogen in LSEC through the NOTCH/HES1/STAT3 pathway. These fibrinogens may foster HSCs activation by binding to integrin αvβ3/5 on HSCs’ membranes, suggesting the POFUT1/NOTCH/HES1/STAT3/fibrinogen axis as a novel therapeutic strategy of liver fibrosis (He et al., 2024). One team has designed a kind of protein named ProAgio targeting integrinαvβ3, to specifically induce apoptosis of activated HSCs and LSECs, which consequently reduces collagen cross-linking, reverses hepatic sinusoidal remodeling as well as mitigates angiogenesis in fibrotic livers (Turaga et al., 2021). Interestingly, there are mechanobiological studies in which a novel perspective was given. During angiogenesis in the early stages of hepatic fibrosis, the mechanical tension generated by the contraction of LSECs was transmitted via collagen, which triggers the DDR2-JAK2/PI3K/AKT-cardiac muscle protein signaling pathway to initiate HSCs activation and ultimately augment the progression of hepatic fibrosis (Liu et al., 2017).

Lipids are deeply involved in the interaction between HSCs and LSECs. Adipocyte fatty acid binding protein (A-FABP), also called aP2 and FABP4, is an adipokine that is secreted into the microenvironment mainly by LSECs in liver fibrosis. In the BDL mouse model, the results of A-FABP knockdown experiments as well as overexpression experiments showed that A-FABP activates the Hh pathway and promotes capillarization of LSECs, as the expression of CD31, a biomarker of capillarization escalated. Meanwhile, A-FABP, in a paracrine manner, stimulates the JNK/c-Jun pathway in HSCs to promote activator protein-1 (AP-1) binding activity. AP-1 interacts with the AP-1 cis-acting sequence in the TGF-β1 promoter to stimulate the transactivation of TGF-β1 in HSCs, which further perpetuates the activation of HSCs (Wu et al., 2021). Since capillarization of LSECs matters in the fibrotic process in a variety of liver diseases, adding the circulating level of A-FABP is positively correlated with the degree of fibrosis in patients with NASH (Furuta et al., 2020), A-FABP is expected to serve as a potent target for inhibiting the fibrogenic pathological process of liver diseases (Wu et al., 2021).

Sphingosine-1-phosphate (S1P) participates in the physiological processes of inflammation, angiogenesis, and regulation of vascular permeability (Hisano and Hla, 2019). It has been exhibited that the sphingosine kinase/S1P/S1PR axis was involved in hepatic angiogenesis, the inhibition of which significantly decreases the mRNA levels of angiogenic markers (Ang1, CD31, VCAM-1) *in vivo* liver fibrosis (Yang et al., 2013). Like hepatocytes, LSECs are able to regulate HSCs activity through exosomes (Sung et al., 2018). LESCs can secrete exosomes within which phospho-sphingosine kinase 1 (SphK1), causes an increase of S1P in exosomes (Wang et al., 2015). S1P is involved in a wide range of cells involved in liver lesions, playing multiple roles in the hepatic microenvironment. TGF-β can mediate the upregulation of SphK1 in fibroblasts and thus elevate S1P levels (Yamanaka et al., 2004). It has been shown that after the internalization of exosomes by HSCs, S1P/SphK1 agonizes pAKT signaling and promotes HSCs activation. In contrast, the activating effects of exosome was attenuated after the use of S1PR2 receptor inhibitors (Barrow et al., 2023). Salidroside (Sal), a phenolic compound presented in the Rhodiola rosea plant, has been reported to have efficacy in a wide range of liver fibrosis. Sal inhibits the expression of SphK1 in serum exosomes as well as HSCs migration induced. In reports where LSECs secreted high levels of SphK1 in exosomes in CCl4-induced hepatic fibrosis, facts were demonstrated that Sal was able to hamper LX-2 activation by SphK1 in exosomes via blocking the activation of the AKT pathway (Ye et al., 2021). Moreover, there are reports that inhibition and silencing of SphK1 can inhibit HSCs activation. In a SphK1 knockout mouse model, less CCL2 is secreted by macrophages and miR-19b-3p is upregulated in HSCs, leading to a corresponding decrease of CCR2 in HSCs membranes by targeting CCR2 (Lan et al., 2018). Similarly, in a mouse model of ischemia-reperfusion in the liver *in vitro*, exosomes deliver SK2 to hepatocytes, resulting in intracellular production of S1P and promoting cell proliferation. However, exosomes from KCs and LSECs lack the same pro-proliferative effect (Nojima et al., 2016). Conversely, S1PR2 on LSECs was able to stimulate TGF-β expression via the YAP pathway, and HSCs were activated by paracrine TGF-β (Liao et al., 2023).

### 5.2 Fibroblast subpopulations

There are different HSCs subgroups in the liver. With gene expression and spatial heterogeneity, they interact and cooperate with each other in generating hepatic ECM. Among the genes specifically expressed by HSCs, the expression of Glypican-3 (GPC3) and DBH genes are mutually exclusive. According to the expression of different specific genes, HSCs are divided into two main subgroups, one is HSC1 (GPC3+), which is mainly confined to the area of the portal vein and central vein, expressing genes such as Neurotrophic Tyrosine Kinase, Receptor, Type 2(NTRK2). The other is HSC2 (DBH+), which is diffusely distributed around the hepatic sinusoids and mainly expresses genes responsible for antigen presentation. However, the presence of a GPC3-DBH- subpopulation of HSCs should also be noted (Payen et al., 2021). Depending on the selection of marker genes, HSCs can also be divided into different subgroups. In other studies, HSCs have been identified into three subgroups (Fred et al., 2022). For example, using Ngfe and Adamtsl2 genes, HSCs are divided into central vein-associated HSCs (CaHSCs) and portal vein-associated HSCs (PaHSCs), which are considered to be the main HSCs producing ECM under CCl4-induced hepatotoxicity and cholestatic liver injury, respectively (Xiong et al., 2019; Dobie et al., 2019; Rosenthal et al., 2021). The spatially specific distribution of HSCs subgroups has reached a consensus, despite the differences in the genes used to categorize them. In the context of liver disease, different subpopulations of HSCs show different activity characteristics as the disease progresses, allowing for the analysis of additional subpopulations such as proliferative (pHSCs), inflammatory (iHSCs), intermediate activation/vascular (vHSCs), contractile/migratory (cmHSCs), and fibrotic myofibroblasts (myHSCs) (Xiong et al., 2019; Rosenthal et al., 2021; Yang et al., 2021; Zhang W. et al., 2021; Krenkel et al., 2019) (Table 1). Yet, there is no universal subpopulation classification with specificity. In addition, lysophosphatidic acid receptor 1 (LPAR1) has been identified as a therapeutic target in collagen-producing HSCs (Dobie et al., 2019).

**TABLE 1:** The heterogeneity of HSCs with the different characters and functions.

Classification criteria	HSC subtypes		Express genes	Functions
Specific expressed gene	HSC1, GPC3+ (Yang et al., 2021)		DBH,HLA-DRB1, HLA-DRA, CD74, HHIP, VIPR1, PTH1R, RAMP1, EDNRB, AGTR1A	Participate in glycosaminoglycan metabolism
	HSC2, DBH+ (Yang et al., 2021)		GPC3, NTRK2, NTRK, EFEMP1, GEM, CCL2, THBS1	Antigen presentation, Hedgehog signal regulation
Specific spatial distribution	Central vein associated HSC (CaHSC) (Krenkel et al., 2019; Lavie et al., 2022)		Adamtsl2, RSPO3, Spon2, Sox4, LoxL1	Located in the center of the lobule that deposit ECM in the fibrotic mouse model
	HSCs associated with portal vein (PaHSC) (Krenkel et al., 2019; Lavie et al., 2022)		NGFR, ITGB3, Igfbp3, IL34, Rgs4	Located in thin wall tissue at the distal end of the fibrotic area, showing proliferative response, but does not transform into collagen-producing cells
Dynamic process of liver fibrosis	Quiescent HSC(qHSC)		Lrat, Rgs5, Ecm1, Angptl6, Vipr1, Gucy1a1, Gucy1b1, Ngfr, Hspa1a, Hspa1b	Maintain normal functions such as vascular tension and relaxation
	Activated HSC (aHSC) (Kostallari et al., 2022; Khan et al., 2024; Lavie et al., 2022)	Proliferation aHSC (pHSC)	Cdc20, cdk1	Cell proliferation
		Inflammation/Immune aHSC (iHSC)	Ccl2, Cxcl1, Cxcl2, Cxcl10, Ccl7, Cd36, Ly6c, CLEC	Stimulate inflammatory and immune responses
		Centre aHSC	IRF7	Present a moderate or lower activation state
		Contractive/Migrated HSC(cm/HSC)	Acta2, Tipm1, vimentin (Vim), Tagln, and tenascin C (Tnc)、Fgl2, Fhl2, Serpin f1, Meg3, Mapf4, Tnnt2, Casq2, Myh11, Myh9, Cnn1	Promote cell migration and contraction
		Fibrotic muscle fibroblasts (myHSC)	Col1a1, Lox, Lum, Clec3b, Mfap5, Pi16, Sfrp1, Fbln1, Mgp, Gsn, Lgfbp6, Nbl1	Promote the production of fibrocollagen and deposition of ECM
	Inactivated HSC		Smoc2, Gabra3, Gsn	

In liver fibrosis, different parts of the liver have different characteristics during the pathologic process due to the spatially differential distribution of different HSCs subpopulations. For example, in the course of liver fibrosis, the phenomenon of uneven distribution of stiff regions of the liver, called stiffness heterogeneity (Kostallari et al., 2022). In the Adamtsl2+ HSCs subpopulation responsible for stiffness heterogeneity, focal adhesion genes (including FHL2) are particularly upregulated due to increased stiffness, promoting HSCs activation (Kostallari et al., 2022). A team labeled HSCs in the periportal region of the hepatic lobule as zone 1 HSCs using SMMHC-CreERT2. In the liver fibrosis model, zone 1 HSCs did not transform into α-SMA-expressing myofibroblasts but were involved in the capillarization of LSECs, suggesting that HSCs subpopulations distributed in different regions are involved in different physiological and biochemical responses (Khan et al., 2024). One study analyzed the HSCs’ secretome genes and found that HSCs can secrete “stellakines” to function as an autocrine/paracrine traffic hub. At the same time, HSCs express a large number of membrane receptors, and several vasoactive hormones interact with G-coupled receptors on the HSCs cell membrane to regulate HSCs contraction (Xiong et al., 2019).

In HCC, the heterogeneity of HSCs has received a lot of attention. Cancer-associated fibroblast (CAF), mainly derived from HSCs, is the most abundant and critical component of the tumor microenvironment (TME), capable of regulating dynamic and complex pathways. CAFs have different subpopulations, ECM remodeling/myofibroblast CAF, pro-inflammatory CAF, immune-regulatory CAF, and antigen-presenting CAF (Lavie et al., 2022). Through ligand-receptor interactions, release of growth factors and inflammatory cytokines, and deposition of ECM components, CAFs are able to directly stimulate cancer cell proliferation or indirectly contribute to tumor development by promoting angiogenesis and remodeling of the microenvironment. iHSCs subpopulation in intrahepatic cholangiocarcinoma (iCCA) secretes HGF, which promotes tumor growth, while in another model of iCCA, myofibroblast CAF subpopulation can secrete type I collagen, which contributes to tumor stiffness without affecting tumor growth; however, myCAF also secretes hyaluronidase 2 (HA2), which promotes the growth and development of iCCA without affecting the stiffness of ECM (Affo et al., 2021). Similarly, studies have focused on cyHSCs, which are enriched with cytokines and growth factors such as the HGF pathway, and HSCs, which are enriched with type I collagen-rich myofibroblasts, in hepatocellular fibrosis. cyHSCs are negatively correlated with myHSCs, with a decrease in cyHSCs and a corresponding increase in myHSCs. myHSCs promote HCC progression, whereas cyHSCs are able to secrete HGF to inhibit tumorigenesis. The balance between cyHSCs and myHSCs during the pathological process influences hepatocellular carcinogenesis (Filliol et al., 2022) (Table 1).

Corresponding to HSCs heterogeneity, the scRNA-seq technique likewise revealed the heterogeneity of hepatocytes, intrahepatic macrophages as well as cholangiocytes (Andrews et al., 2022), giving a more promising outlook for the study of liver diseases (Cheng et al., 2020). Although the heterogeneity of various cells in different pathological conditions of the liver has been studied intensively, the crosstalk within subpopulations and between each subpopulation and other cells still needs to be further explored.

## 6 Conclusions and future perspectives

Current understanding suggests that liver fibrosis and early cirrhosis may be reversible. Therefore, the study of liver fibrosis has emerged as a prominent topic within the field of liver disease. Considering the liver is a complex organ containing various cell types, including hepatocytes, LSECs, hepatic macrophages, HSCs, and other immune cells, the interactions among various cell types must be taken seriously when exploring the influence and mechanisms underlying liver fibrosis. While on this basis, the primary cells responsible for triggering liver fibrosis may vary when specific factors inducing liver injury, leading to different responses mediated by associated genes. Therefore, different targeted treatment and prevention strategies should be adopted for the primary cells that cause liver fibrosis due to different reasons, and the time-dependent therapeutic strategies depended on the primary cells responsible for triggering liver fibrosis should also be considered.

## Glossary

HSC: Hepatic stellate cell
LSECs: Liver sinusoidal endothelial cells
MMP: Matrix metalloproteinases
ILTC: Innate lymphocyte populations
P2Y14: Purinergic Receptor P2Y14
TGF-β: Transforming growth factor-β
OPN: Osteopontin
EN: Endosialin
MdM: Monocyte-derived macrophage
MASH: Metabolic dysfunction-associated steatohepatitis
LAM: Lipid-associated macrophage
CTGF: Connective tissue growth factor
ECM: Extracellular matrix
BMDMs: Bone marrow-derived macrophages
CCL2: Chemokine C-C motif ligand 2
MAPK: Mitogen-activated protein kinase
NLRP3: NOD-like receptor family pyrin domain containing 3
MAIT: Mucosal-Associated Invariant T cell in liver diseases
GARP: Glycoprotein A repetitions predominant
A-FABP: Adipocyte Fatty Acid Binding Protein
S1P: Sphingosine-1-phosphate
SphK1: Sphingosine Kinase 1
AIRN: Antisense Of IGF2R Non-Protein Coding RNA
KLF2: Kruppel-like factor 2
POFUT1: Protein O-fucosyltransferase
Zeb2: Zinc-finger E-box-binding homeobox2
IGF1: Insulin-like growth factor-1
DAMPs: Damage-associated molecular modules
HMGB1: High Mobility Group Box-1
NTCP: Sodium+/taurocholate cotransporting polypeptide
HCC: Hepatocyte carcinoma
EV: Extracellular vesicles
NASH: Non-alcoholic steatohepatitis
NAFLD: Non-alcoholic fatty liver disease
FoxO1: Forkhead transcription factor 1
EMT: Epithelial-mesenchymal transition
E4BP4: E4-binding protein 4
MDR2: Multidrug resistance protein 2
EZH2: Enhancer of zeste homologue 2
CAF: Cancer-associated fibroblasts
iCCA: Intrahepatic cholangiocarcinoma
MCP-1: Monocyte chemotactic protein-1
Fn: Fibronectin
CCL: C-C Motif Chemokine Ligand
Gpnmb: Glycoprotein NMB
PD-L1: Programmed death ligand 1
TLR: Toll like receptor
DNTs: Double-negative T-cells
CAR: Chimeric antigen receptor
HGF: Hepatocyte growth factor
